# Cloning and characterization of *MAP2191* gene, a mammalian cell entry antigen of *Mycobacterium avium* subspecies *paratuberculosis*

**DOI:** 10.22099/mbrc.2018.30979.1354

**Published:** 2018-12

**Authors:** Zahra Hemati, Masoud Haghkhah, Abdollah Derakhshandeh, ShoorVir Singh, Kundan Kumar Chaubey

**Affiliations:** 1 Department of Pathobiology, School of Veterinary Medicine, Shiraz University, Shiraz, Iran; 2 Department of Biotechnology, Institute of Applied Sciences and Humanities, GLA University, Chaumuhan, Mathura, UP, India 281406

**Keywords:** Johne's disease, Control, mce gene, MAP2191, Hydrophobicity

## Abstract

The aim of this study is to identify, clone and express a *Mycobacterium avium *subsp.* paratuberculosis *specific immunogenic antigen candidate, in order to develop better reagents for diagnosis and vaccines for the protection of the host. Therefore, *MAP2191* gene (a member of MAP*mce*5 operon) from MAP, was isolated and characterized by Bioinformatics tools and *in vitro* experiments. Then, a novel Mce-whole protein encoded by *MAP2191* gene was amplified and sub-cloned into *E. coli*. We tried to express the Mce/whole protein in different condition along with a positive expression control (pET28a-Mce/truncated plasmid that we know express well), to ensure that nothing is wrong regarding culture/induction condition. The level of the recombinant protein expression was analyzed by means of SDS-PAGE and Western blotting. Western blot analysis toward full-length MAP2191 protein and its truncation only demonstrated Mce/truncated protein. The concurrence of *in-silico* prediction of primary structure of MAP2191 protein results along with experimental results confirmed that expression of Mce/whole protein was affected by the hydrophobicity nature of this protein. Our data support the hypothesis that the presence of hydrophobic regions in protein structure can influence the level of recombinant protein expression. This stresses the importance of gene selection and the protein sequence checking of the hydrophobic content in any protein purification project in order to achieve a large amount of desirable proteins.

## INTRODUCTION

Johne’s disease (JD) or paratuberculosis is a chronic infection of the intestinal tract of animals, which is caused by *Mycobacterium avium *subsp.* paratuberculosis *(MAP) [1, 2]. JD is primarily a disease of ruminants (cattle, sheep, goats, buffaloes etc.), but can affect non-ruminants, especially wildlife [3, 4]. High to very high prevalence of JD has been frequently reported from dairy farms world-wide [5, 6]. JD is recognized as an important public health pathogen duo to the presence of live MAP bacilli in animal milk (both raw and pasteurized) and other dairy products [1,2,7]. There are potential association of this multi-host pathogen with human diseases like Crohn’s disease and several other chronic inflammatory syndromes [8, 9]. Thus, current trends in diagnosis and disease control of JD are searching for identification and characterization specific immunogenic antigen candidates of MAP [10, 11].

Mammalian cell entry (Mce) protein was first recognized in *Mycobacterium tuberculosis *(MTB) and was shown have a possible role in the virulence of MTB, which allows non-pathogenic *Escherichia coli* to enter into non-phagocytic cells [13-15]. Although *Mce *genes were originally identified in MTB, homologous of these genes are widely distributed throughout the *Mycobacterium *genus [16-18]. Mce proteins were characterized as invasion‐like proteins localized at the cell surface of the mycobacteria [19]. Eight homologous regions of *mce *gene families have predicted to be located in the outer membrane of all the MAP isolates [20]. In MAP K-10, *mce*5 operon included a cluster of six homologous genes (*MAP2189*-*MAP2194*) that predicted to encode proteins involved in lipid uptake [21, 22]. These *mce5* genes play important roles in MAP invasion, survival and virulence [22]. *MAP2191* gene is a member MAP*mce*5 operon, having potential antigenic nature and immunogenic T- and B-cell epitopes specific for MAP based on our in*-silico* analyses. This prompted us to perform an antigen discovery study with the objective of characterizing full length of *MAP2191* gene both at genome and protein level using cloning, gene expression techniques and bioinformatic analysis. In this study, we tried to express the Mce/whole protein and discovered some interesting trends that might be appreciated by future investigators in their recombinant protein purification studies.

## MATERIALS AND METHODS

**Morphological and molecular characterization of MAP: **MAP reference strain ‘S 5’ Indian Bison Type (provided by Central Institute for Research on Goats (CIRG), Makhdoom, Mathura, Uttar Prades in India), was grown on the slant of Herold’s egg yolk agar (HEY) medium as per Singh et al., [23]. Molecular confirmation of the strain was done by IS*900 *[24], IS*1311 *PCR [25] and IS*1311* PCR-REA [26]. All primers sequences are listed in Table 1.

**Table 1:** Primers used for characterization of *Mycobacterium avium* subsp. *paratuberculosis*

**Target**	**Primer**	**Primer Sequence**	**Product size (bp)**
**IS** ***900***	P90	5’ GAAGGGTGTTCGGGGCCGTCGCTTAGG 3’	413
	P91	5’ GGCGTTGAGGTCGATCGCCCACGTGAC 3’	
**IS** ***1311***	M56	5’ GCGTGAGGCTCTGTGGTGAA 3’	608
	M119	5’ ATGACGACCGCTTGGGAGAC 3’	
**MAP2191**	F	5’ACTGGATCCCTGAAATACCGTGGCGCAAAC 3’- *Bam*H1	1065
	R	5’CTAAAGCTTTCATCGCGAACCGCCCGGGATG 3’– *Hin*dIII	

**In** ***-silico*** **analyses of MAP** ***2191*** **gene and protein identification: **In*-silico* analysis of the MAP2191 protein sequence was carried out using the basic local alignment search (BLASTp) analysis and SOPMA web site. Mce/whole protein coding sequence (MAP2191 full length protein referred by this short name) was subjected to BLASTp analysis at the NCBI GenBank site to confirm the MAP sequence identity. The antigenicity and hydrophobicity analysis of the Mce/whole protein molecule were also done using the CLC Genomics Workbench 7.5.1 program (CLC bioQIAGEN, Germany) software.

**Preparation of full-length version of the** ***MAP2191*** **gene by PCR: **Full length of the *MAP2191* gene, namely *mce*/whole gene successfully amplified with primers were designed to have *Bam*HI (forward primer) and *Hin*dIII (reverse primer) restriction enzyme sites as depicted in Table 1. PCR master mix (2X) (Cat no. #K0171, Thermo Scientific) containing all components of the reaction mixture (dNTPs, Taq DNA polymerase, 10X PCR buffer, MgCl_2_ and loading dye) were used. Positive control (containing DNA from the reference strain as template) and negative control (containing nuclease free water) are also prepared along with the test DNA samples. The PCR products was confirmed by nucleic acid sequencing.

**Cloning of** ***mce*** **-whole gene in TA-cloning vector: **The confirmed and purified gene segment of respective *mce*/whole gene was cloned in pTZ57R/T cloning vector using ligase enzyme (Fermentas, USA), as per manufacturer’s instructions. The ligation product (pTZ57R/T plasmids carrying *mce/*whole gene) was successfully transformed with heat shock at 42^◦^C for 90 Sec into *E.coli *XL10-Gold ultra-competent cell (Stratagene, Agilent).White transformed *E.coli* colonies were selected and further screened by colony PCR with *mce*/whole primers for the selection of recombinant clones.Final confirmations of selected plasmids were done using restriction digestion followed by DNA sequencing.

**Sub-cloning of** ***mce*** **-whole gene in expression vector: **Expression vector was prepared by ligation of *Bam*H1/*Hin*dIII digested, de-phosphorylated (by the fast alkaline phosphatase enzyme, Cat. No: #EF0651, Fermentas, USA), and gel purified pET28a and *Bam*H1/*Hin*dIII digested and gel purified *mce*/whole gene fragment (insert) by using ligase enzyme (Fermentas, USA). Specific product was cut from the gel and the gel cut was purified using agarose gel DNA purification Kit (Thermo scientific, USA). Colonies were confirmed for the presence of the insert by colony PCR followed by double digestion. One of the confirmed pET28a-*mce*/whole vectors was sequenced using T7 universal primers for further confirmation.

**Expression and purification of recombinant Mce-whole protein: **Sequences-confirmed pET28a-*mce*/whole vector was used to transform *E. coli* Rosetta (Novagene, WI, USA) and *E. coli* BL-21 (DE3, Novagene, WI, USA) competent cells. The expression of the transformed cloned was induced by adding different concentrations of isopropyl-β-D thiogalactopyranoside (IPTG) (0.1-1.5 mM) at four different temperatures (37°C, 30°C, 23°C and 16°C) and different growth time intervals (2, 4, 8, 12, 16, 24, 30 hours). Each collected samples were further analyzed by 12% sodium dodecyl sulfate polyacrylamide gel electrophoresis (SDS-PAGE) to enquired expected size of the differently expressed Mce-whole protein in the different IPTG induced time point samples versus the un-induced sample. Sonicated extracts (crude, supernatant and pellets) of IPTG induced recombinant *E. coli* strains containing pET28a-*mce*/whole plasmid was also separated by SDS-PAGE and analyzed for the presence of Mce/whole gene by western blotting. To test the protein expression and purification efficiency, Mce/truncated protein co-purified along with Mce/whole protein in our experiment as a positive expression control.

## RESULTS

**Figure 1:**
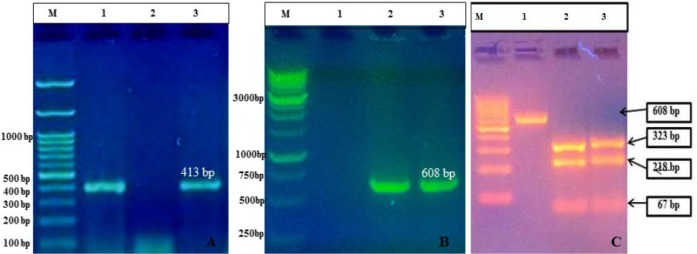
Molecular Characterization of MAP ‘S5’ DNA: (A) IS*900* PCR: lane M- 100 base Marker (#SM0243, Fermentas), lanes 1 and 3: IS*900* PCR products; (B) IS*1311* PCR: lane M: 1KB Marker (#MBT51, Hi-Media), lanes2 and 3: IS*1311* PCR products; (C) IS1311-REA genotyping:lane M- 100 base Marker (#SM0243, Fermentas), Lane 1: IS*1311* PCR product,lane 2: Digested PCR product of IS*1311* from Positive control, lane 3: Digested PCR product of IS*1311* from sub-cultured colonies.

MAP reference strain (S5) colonies on HEY slant supplemented with Mycobactin J. were small, translucent, straw color, having nipple over raised part and loosely attached to the surface of the medium. Bacterial cells from MAP colonies were strongly acid-fast on Ziehl-Neelsen staining. The presence of the specific PCR products was considered as positive for MAP with IS*900* PCR (Fig. 1A), IS*1311* PCR (Fig. 1B) and IS*1311* PCR-REA (Fig. 1C).

**Figure 2:**
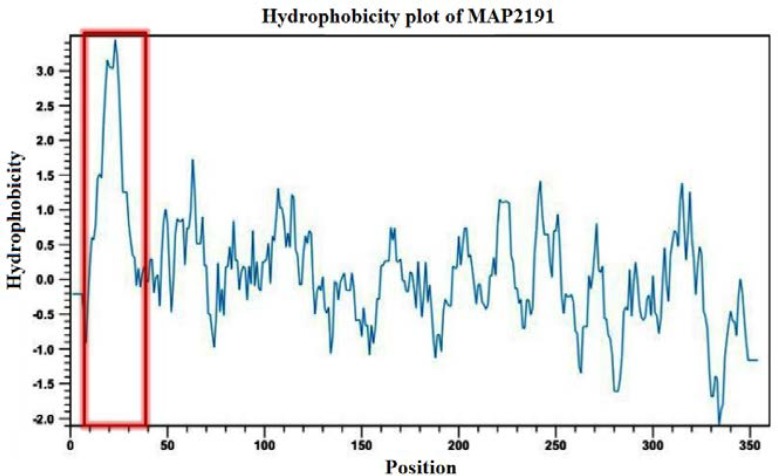
Hydrophobicity analysis of MAP2191 protein. The presence of hydrophobic N-terminal loop is shown in a red box.

According to alignment sequence comparison analysis, *MAP2189-MAP2194 *cluster organization is conserved among MAP K-10 and MAP S 5 strain sequences and most epitopes are shared. The homology analysis also showed the MAP2191 protein was highly specific as unique to MAP. The *MAP2191* gene is 1065bp and has 354 amino acids with an estimated molecular weight of 37.510 kDa, an isoelectric point of 4.9, a net charge of -3.8 at pH 7.0 and good water solubility. Web-based analysis of the full length of MAP2191 protein using SOPMA web site revealed this protein displayed 41.53% α-helix content, 11.58% β-turn content, 16.95% extended strand and 29.94% random coil structures. CLC Genomics Workbench software analysis predicted the presence of a highly hydrophobic loop in N-terminal amino acid sequence of the full length MAP2191 protein from residues 10 to 32 (Fig. 2). In addition, the C-terminal portion of MAP2191 protein was more hydrophilic than N-terminal portion and had a high antigenic index as shown in*-silico *analysis results.

The *mce*/whole gene was successfully amplified from MAP ‘S 5’ DNA by PCR. A band of approximately 1083bp (1065 *mce* gene and extra 18bp used to bring the coding sequence in-frame and for restriction enzyme sites) was considerate as positive for the whole gene. PCR product’s sequencing showed 100% homology of *mce*/whole nucleotide sequence with *MAP2191* gene sequences present in the complete genome sequence of MAP K10 available in the GenBank (Accession number: NC_002944.2).

**Figure 3:**
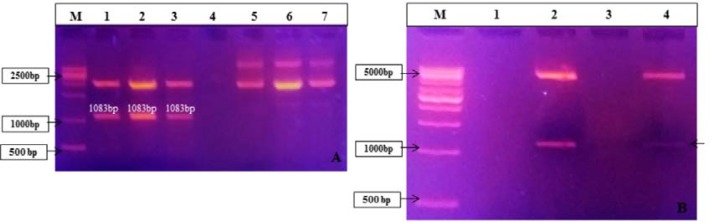
(A) Restriction digestion of confirmed colony PCR positive pZ57R/T *mce*-whole clones: lane M: 1kb DNA ladder (#SM0313, Fermentas), lanes 1, 2 and 3: Positive clones of PTZ57R/T *mce*-whole plasmid digested with *Bam*HI and *Hin*dIII enzymes; Lanes 5, 6 and 7: Plasmid isolated of positive PTZ57R/T *mce*-whole clones. (B) Restriction Digestion of confirmed colony PCR positive pET28a *mce*-whole clones; lane M: 1kb DNA ladder (#SM0313, Fermentas),lanes 2 and 4: Positive clones of pET28a *mce*-whole plasmid.

The present of *mce*-whole gene segment (insert) in the TA cloning vector and in the pET28a expression vector was confirmed using specific colony PCR and restriction analysis (Figs. 3). Gene sequencing also confirmed the positive *mce*-whole-pTZ57R/T and *mce*-whole-pET28a clones.

**Figure 4:**
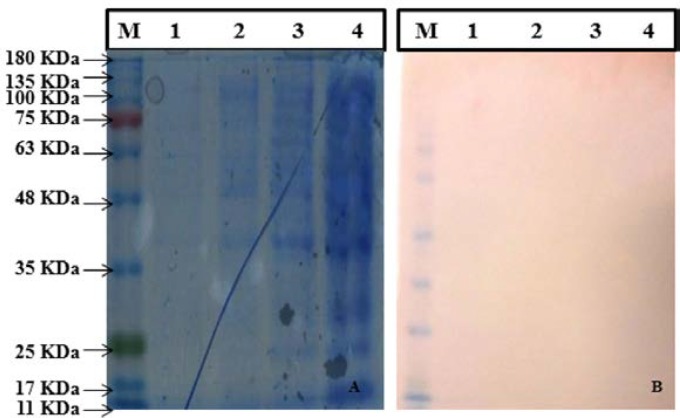
(A) Analysis of Mce-whole Protein expressed by SDS PAGE. (Left to Right), lane M: Protein molecular weight marker (SL7012, CinnaGen), lane 1: pET28a.Mce.whole Elution 1, lane 2: pET28a.Mce.whole Supernatant, lane 3: pET28a.Mce.whole Pellet, lane 4. pET28a.Mce.whole Crude; (B) Western blot analysis (from left to right) lane M: Protein molecular weight marker (SL7012, CinnaGen), lane 1: pET28a.Mce.whole Elution 1, lane 2: p1ET28a.Mce.whole Supernatant, lane 3: pET28a.Mce.whole Pellet, lane 4. pET28a.Mce.whole Crude.

SDS-PAGE results indicated that there was no obvious expression of Mce/whole protein in all fractions collected from different IPTG concentration and different time point samples.Although, no band at the same size of Mce/whole protein was detected in running the crude, supernatant and pellets of sonicated cells on an SDS-PAGE gel (Fig. 4A). No such band was obtained with sonicated extracts of IPTG-induced *E. coli* strains harboring pET28a-*mce* plasmid by Anti-His-Tag monoclonal antibody (Fig. 4B).

## DISCUSSION

Johne’s disease (JD) in animals presents as a chronic disease characterized by enteritis, weakness, diarrhea, emaciation and death [27]. Diagnosis, control and laboratory confirmation of MAP is always challenging [1, 4]. Mammalian cell entry (*mce*) genes are known to be virulence factors involved in mycobacterial entry and survival within macrophages and can facilitate host invasiveness [12, 28, 29]. In the present study the *MAP2191* gene (a member of MAP*mce*5 operon) from MAP, was isolated and characterized by Bioinformatics tools and in vitro experiments. Bioinformatics analysis results revealed the protein’s amino acids 10-32 formed a hydrophobic loop at the N-terminal end of full-length MAP2191 protein and the presence of such hydrophobic region can block expression of the Mce/whole protein.

Although a lot of protein expression systems have been developed today, but achieving a pure protein is still a problematic duo to the presence of labor-intensive cloning, expression and purification steps [30]. In our study, expression of full-length MAP2191 protein was performed similar to the cloning and expression strategies used to produce recombinant Mce/truncated protein, but no expression of Mce/whole protein was observed in induced *E. coli* cell containing pET28a-*mce*/whole plasmid on SDS-PAGE at different IPTG-concentration and temperatures. The level of expression of the Mce/whole maybe being too less to be detectable on gel, so we have also gone for protein specific antibody where we could say there is no complete expression. Western blot analysis toward full-length MAP2191 protein and its truncation only demonstrated Mce/truncated protein, which was similar to that predicted for Mce/whole protein.

Two usual concepts that will result in very low or no yield of protein during protein purification studies are protein toxicity and codon bias [31]. We were sure that our expression vector is correct and there was no mutation in the sequence based on sequencing results. If Mce/whole protein happened to be toxic for *E. coli*, we should see that transformed *E. coli* strain grow slower than the simple vector transformed strain in liquid culture [44]. In the case of protein proteolytic degradation problem, we tried to express the full-length protein in another *E. coli* strain like Rosetta expression cell in soluble condition with the pET28a vector system. Since, anti-His-Tag monoclonal antibody could not detect any Mce/whole protein from either Bl-21 or Rosetta strains and the results were the same when we used of Rosetta strain as an alternative host.We checked for our specific protein in cell lysate, supernatant and pellet also, because some protein is insoluble, which will present in pellet, but still didn’t get expression in any of the fractions. Therefore, in-*silico* prediction of primary structure of MAP2191 protein results along with experimental results confirmed that expression of Mce/whole protein was affected by the hydrophobicity nature of this protein. Our results were exactly in agreement with those of Cho et al., (2007), who described cloning and expression of C- and N-terminal portion of PepA protein separately and recorded only C-terminal end was successfully expressed due to the presence of hydrophilic eras [32]. In their study also N-terminal portion of PepA protein was predicted to be more hydrophobic than the C-terminal portion based on in*-silico *analysis. Lu et al., (2006), also reported a short sequence of 22 amino acids, the most hydrophilic region of Mce1A protein, is the only functional domain of this protein [14]. Strych et al., (2001), also reported that the presence of N-terminal hydrophobic region may interfere with Alr protein dimerization, folding or ligand binding [33].

In summary, our study results showed that the presence of hydrophobic structure, especially in first initialing amino acids in the protein’s chain can influence the protein expression system. Our data provide a valuable guide for future investigators with their own protein expression project to scan the protein sequence for the hydrophobic region in the initiating steps of protein purification to have an idea about final expected protein yield and to make more informed choices.
